# Metal Complexes of the Porphyrin-Functionalized Polybenzoxazine

**DOI:** 10.3390/polym14030449

**Published:** 2022-01-23

**Authors:** Guohu Zhang, Ahmed F. M. EL-Mahdy, Lamiaa Reda Ahmed, Babasaheb M. Matsagar, Sameerah Al-Saeedi, Shiao-Wei Kuo, Kevin C.-W. Wu

**Affiliations:** 1 Center for Functional Polymers and Supramolecular Materials, Department of Materials and Optoelectronic Science, National Sun Yat-Sen University, Kaohsiung 80424, Taiwan; m083100049@student.nsysu.edu.tw (G.Z.); ahmedelmahdy@mail.nsysu.edu.tw (A.F.M.E.-M.); d102060004@student.nsysu.edu.tw (L.R.A.); 2 Institute of Medical Science and Technology, National Sun Yat-Sen University, Kaohsiung 80424, Taiwan; 3 Department of Chemical Engineering, National Taiwan University, No. 1, Sec. 4, Roosevelt Road, Taipei 10617, Taiwan; matsagar03@ntu.edu.tw; 4 Department of Chemistry, College of Science, Princess Nourah Bint Abdulrahman University, Riyadh 11671, Saudi Arabia; sialsaeedi@pnu.edu.sa; 5 Department of Medicinal and Applied Chemistry, Kaohsiung Medical University, Kaohsiung 80708, Taiwan; 6 International Graduate Program of Molecular Science and Technology, National Taiwan University (NTU-MST), No. 1, Sec. 4, Roosevelt Road, Taipei 10617, Taiwan

**Keywords:** polybenzoxazine, ring-opening polymerization, porphyrin, metal complex, thermal stability

## Abstract

New porphyrin-functionalized benzoxazine (Por-BZ) in high purity and yield was synthesized in this study based on ^1^H and ^13^C NMR and FTIR spectroscopic analyses through the reduction of Schiff base formed from tetrakis(4-aminophenyl)porphyrin (TAPP) and salicylaldehyde and the subsequent reaction with CH_2_O. Thermal properties of the product formed through ring-opening polymerization (ROP) of Por-BZ were measured using DSC, TGA and FTIR spectroscopy. Because of the rigid structure of the porphyrin moiety appended to the benzoxazine unit, the temperature required for ROP (314 °C) was higher than the typical Pa-type benzoxazine monomer (ca. 260 °C); furthermore, poly(Por-BZ) possessed a high thermal decomposition temperature (*T*_d10_ = 478 °C) and char yield (66 wt%) after thermal polymerization at 240 °C. An investigation of the thermal and luminescence properties of metal–porphyrin complexes revealed that the insertion of Ni and Zn ions decreased the thermal ROP temperatures of the Por-BZ/Ni and Por-BZ/Zn complexes significantly, to 241 and 231 °C, respectively. The metal ions acted as the effective promoter and catalyst for the thermal polymerization of the Por-BZ monomer, and also improved the thermal stabilities after thermal polymerization.

## 1. Introduction

The chemistry of benzoxazines (BZs) has attracted much interest for two decades now, owing to their potential applications in coatings, composites, and electronics [1,2,3,4,5,6]. The thermal ring-opening polymerizations (ROPs) of BZ monomers result in highly cross-linked structures, formed as a result of Mannich condensations of phenolic and formaldehyde derivatives with primary amines [7,8,9,10,11,12,13]. Because strong inter- and intramolecular hydrogen bonds exist after the thermal ROP of BZ units, formed among the tertiary amino and phenolic units in the Mannich bridges, the polymerization products can possess low dielectric constants, low surface free energies, low degrees of shrinkage, and high thermal stabilities and char yields [14,15,16,17,18,19,20]. Flexibility in molecular design—by varying the structures of the phenolic and amino groups—has allowed the introduction of a range of functional groups (e.g., allyl, propargyl, crown ether) [21,22,23,24,25,26,27,28,29,30,31] or inorganic nanomaterials (such as polyhedral oligomeric silsesquioxane, graphene, carbon nanotube) [32,33,34,35,36,37,38,39,40,41] into the BZ matrix.

Porphyrin is a molecular material with many potential applications; for example, because of its unique magnetic, photophysical, and electronic properties, it has been integrated in light–energy conversion systems [42,43,44,45]. This colored macrocycle possesses particularly attractive photophysical properties, including very high extinction coefficients in the near-IR and visible regions where the solar photon flux occurs, making it useful in redox chemistry, photo-harvesting, semiconductors, and photoinduced electron transfer [46,47]. Various porphyrin-functionalized polymers have been prepared, usually connected with metal ions or reactive units [48,49,50]. Based on several previous reports [41,42,43,44,45,46,47,48,49], in this study, the synthesis and ROP of a porphyrin-functionalized benzoxazine (Por-BZ) monomer were investigated; to the best of our knowledge, such structures have not been described previously.

**Scheme 1:**
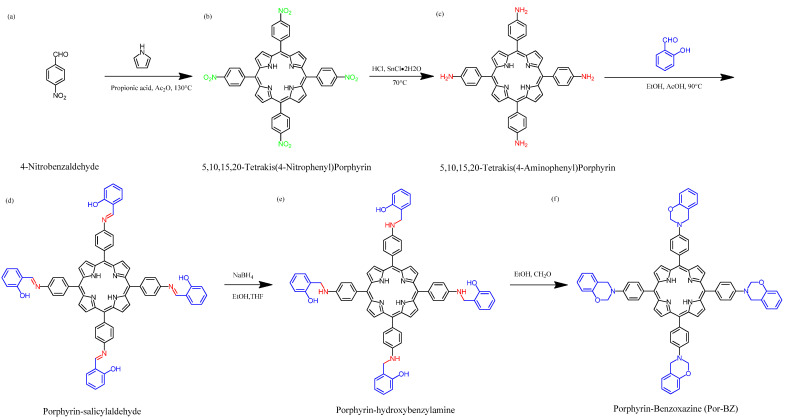
Synthesis of (**f**) Por-BZ monomer from (**a**) 4-nitrobenzaldehyde, (**b**) TNPP, (**c**) TAPP, (**d**) Por-Sa, and (**e**) Por-Hy.

We synthesized Por-BZ through a Schiff base formation from tetrakis(4-aminophenyl)porphyrin (TAPP) and salicylaldehyde and subsequent treatment for *o*-hydroxybenzylamine derivative with CH_2_O (Scheme 1). Fourier transform infrared (FTIR) and nuclear magnetic resonance (NMR) spectroscopy were used to confirm the chemical structure of Por-BZ; we then employed differential scanning calorimetry (DSC), FTIR spectroscopy, and thermogravimetric analysis (TGA) to determine the thermal ROP behavior of Por-BZ and its thermal stability in the presence of Ni and Zn ions. Furthermore, we used UV–Vis and photoluminescence (PL) spectroscopy to examine the luminescence properties of TAPP, Por-BZ, and Por-BZ/metal ion complexes.

## 2. Experimental Section

### 2.1. Materials

Pyrrole (99.5%), 4-Nitrobenzaldehyde (99%), salicylaldehyde (99%), EtOH (99.9%), acetic acid (99.8%), formaldehyde, hexane (99%), and *N*,*N*-dimethylformamide (DMF, 99.5%) were purchased from Acros (Taipei, Taiwan). Propionic acid (99.5%), acetic anhydride (99%), tetrahydrofuran (THF, 99%) MeOH (99%), pyridine (99%), hydrochloric acid, and chloroform (99.9%) were purchased from Sigma-Aldrich (Taipei, Taiwan). SnCl_2_·2H_2_O (99%), zinc acetate dihydrate (99%), and nickel acetate dihydrate (99%) were purchased from Showa (Kaohsiung, Taiwan) and used without purification.

### 2.2. 5,10,15,20-Tetrakis(4-Aminophenyl)Porphyrin (TAPP)

Propionic acid (80 mL), 4-Nitrobenzaldehyde (3.0 g, 0.020 mol), and acetic anhydride (3.2 mL, 0.034 mol) were heated to 130 °C in a three-neck round-bottom flask, and then pyrrole (1.38 mL, 0.020 mol) was added dropwise via syringe. After heating under reflux for 1 h, the mixture was cooled to room temperature to obtain a black precipitate, which was collected by filtration and washed three times with MeOH. The black solid was dissolved in pyridine (100 mL) and heated under reflux at 110 °C for 4 h; the mixture was placed in a refrigerator overnight. The precipitate was collected by filtration and washed three times with MeOH. This product was dried under vacuum at 80 °C for 24 h to give tetrakis(4-nitrophenyl)porphyrin (TNPP) as purple microcrystals (yield: 15%). A solution of TNPP (0.60 g, 0.76 mmol) in concentrated HCl (36.5%, 40 mL) was heated up to 70 °C in a two-neck flask, then SnCl_2_·H_2_O (4.5 g, 200 mmol) was added. The mixture was stirred at 70 °C for 2 h and then cooled to 0 °C. After neutralization with aqueous NH_3_, the gray product was collected by filtration and dissolved in THF. After ultrasonication, the mixture was filtered to remove any insoluble materials and then the filtrate was concentrated (rotary evaporation) and dried under vacuum to yield TAPP as violet crystals (yield: 80%).

### 2.3. Porphyrin-Salicylaldehyde (Por-Sa)

A solution of TAPP (0.50 g, 0.74 mmol) and salicylaldehyde (0.48 mL, 4.5 mmol) in EtOH (70 mL) and acetic acid (99.8%, 1 mL) was heated at 90 °C for 4 h under reflux. The solid was filtered off after cooling, washed with EtOH, and dried under vacuum (70 °C, 12 h) to obtain porphyrin-salicylaldehyde as a purple solid (yield: 57%).

### 2.4. Porphyrin-Hydroxybenzylamine (Por-Hy)

A mixture of Por-Sa (0.80 g, 1.7 mmol), sodium borohydride (300 mg, 6.7 mmol), EtOH (40 mL) and dry THF (16 mL) was stirred in a 100-mL three-neck flask for 24 h at 25 °C. The resulting solution was concentrated (rotary evaporation) until the volume had halved. This dark-green solution was poured into ice water in a 500-mL beaker and then stirred. After filtration and washing with deionized water, the Por-Hy was dried under vacuum (70 °C, 12 h) to provide porphyrin-hydroxybenzylamine (yield: 40%).

### 2.5. Porphyrin-Salicylaldehyde-Benzoxazine (Por-BZ)

A mixture of Por-Hy (0.70 g, 0.64 mmol), THF (10 mL), absolute EtOH (60 mL), and paraformaldehyde (2 mL, 7.3 mmol) was stirred in a 100-mL three-neck flask at 90 °C. Periodically, a drop of the reaction mixture was placed in a small amount of THF and thin layer chromatography (THF/hexane, 1:1) was used to monitor the reaction. After 24 h, the solid was filtered off, washed with EtOH, and dried under vacuum (70 °C, 12 h) to obtain Por-BZ as a purple powder (yield: 85%).

### 2.6. Por-Benzoxazine–Nickel(II) and –Zinc(II) Complexes (Por-BZ/Ni and Por-BZ/Zn)

**Scheme 2:**
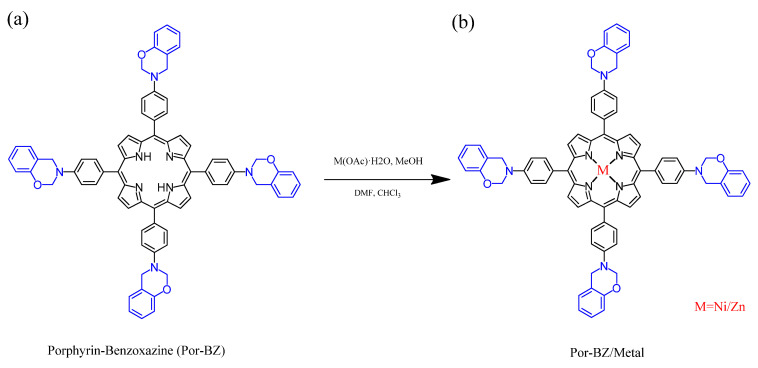
Chemical structures of (**a**) Por-BZ and (**b**) the Por-BZ/metal complexes.

Por-BZ (100 mg, 0.087 mmol), DMF (20 mL), and CHCl_3_ (45 mL) were placed in a flask. Ni(OAc)2·4H_2_O (150 mg, 0.6 mmol) (or Zn(OAc)_2_·2H_2_O (130 mg, 0.6 mmol)) was dissolved in MeOH (20 mL); after ultrasonication, it was added dropwise into the reaction mixture, which was then heated at 60 °C for 24 h. Then, the solution was partitioned between H_2_O and CHCl_3_ after cooling to 25 °C; the organic phase was washed twice with brine, dried, and concentrated (rotary evaporation). The residual DMF was evaporated through vacuum distillation (60–70 °C, 1 h). THF (20 mL) was added to the residue. After rotary evaporation and drying under vacuum (70 °C, 12 h), Por-BZ/Ni (or Por-BZ/Zn) was obtained (yield: 48%) as shown in Scheme 2.

### 2.7. Polymerization of BZ Monomer

**Scheme 3:**
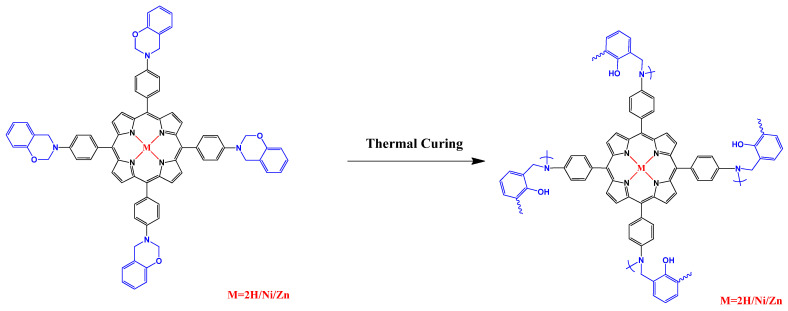
Thermal ring-opening polymerization of the Por-BZ, Por-BZ/Ni, and Por-BZ/Zn.

The Por-BZ monomer underwent thermally ring-opening polymerization by heating at 150, 180, 210, 240, 270, and 300 °C (2 h at each temperature) in a stepwise manner. In the same manner, Por-BZ/Ni and Por-BZ/Zn were also subjected to thermal polymerization by heating at 150, 180, 210, and 240 °C; they were at each temperature for 2 h (Scheme 3).

## 3. Results and Discussion

### 3.1. Synthesis of TAPP

**Figure 1:**
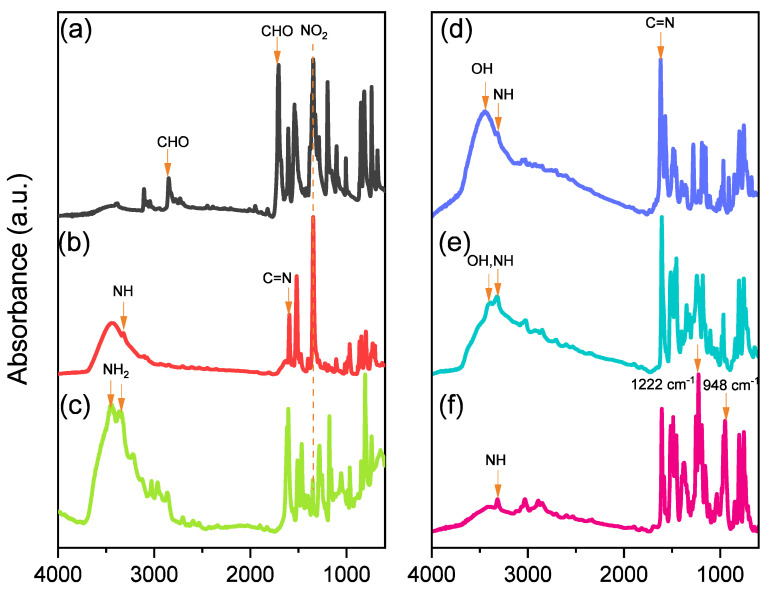
FTIR spectra of (**a**) 4-nitrobenzaldehyde, (**b**) TNPP, (**c**) TAPP, (**d**) Por-Sa, (**e**) Por-Hy, and (**f**) Por-BZ.

**Figure 2:**
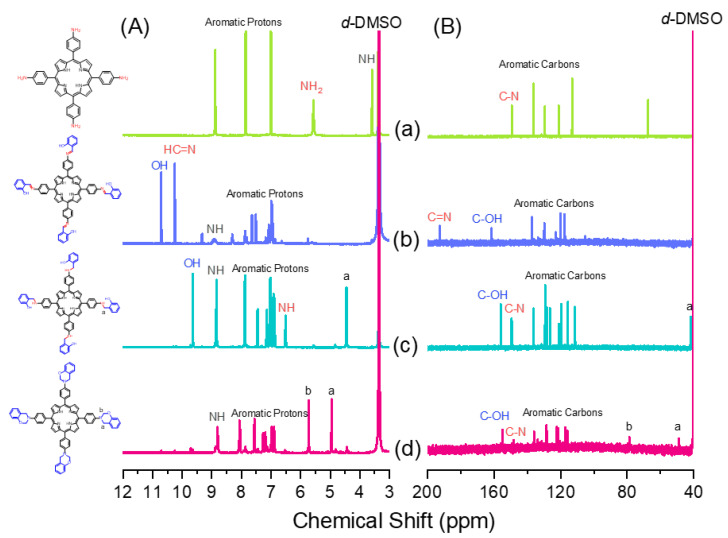
(**A**) ^1^H and (**B**) ^13^C NMR spectra of (**a**) TAPP, (**b**) Por-Sa, (**c**) Por-Hy, and (**d**) Por-BZ.

Scheme 1 presents the synthesis of the monomer Por-BZ from 4-nitrobenzaldehyde (Scheme 1a). First, we prepared TNPP (Scheme 1b) through the reaction of 4-nitrobenzaldehyde with pyrrole in the presence of propionic acid and acetic anhydride at 130 °C. The reduction of TNPP with HCl and SnCl_2_·H_2_O at 70 °C for 2 h provided TAPP as violet microcrystals in high yield and purity (Scheme 1c); FTIR and NMR spectroscopy confirmed the chemical structure. Figure 1a–c display the FTIR spectra of 4-nitrobenzaldehyde, TNPP, and TAPP, respectively, each recorded at room temperature. The signals for the NO_2_ units appeared near 1346 and 1593 cm^−1^ for both 4-nitrobenzaldehyde and TNPP; the signals of the CHO unit of 4-nitrobenzaldehyde at 2855, 2776, 2733 (C–H) and 1700 (C=O) cm^−1^ were absent from the spectrum of TNPP, but signals appeared for an NH unit at 3312 cm^−1^ and a C=N unit at 1596 cm^−1^ [51]. In the spectrum of TAPP, the signals of the NO_2_ units had almost disappeared, with two new absorption peaks appearing for NH_2_ units at 3352 and 3439 cm^−1^. Figure 2 show ^1^H and ^13^C NMR spectra of TAPP. The signals of the NH and NH_2_ protons appeared at 3.57 and 5.56 ppm, respectively (Figure 2A). The signal at 149.32 ppm in Figure 2B represents to the C–N units of TAPP, confirming the successful reduction of TNPP to form TAPP in high purity.

### 3.2. Synthesis of Por-BZ

Although TAPP features four amino groups, one-pot Mannich condensations from primary amines, phenol, and CH_2_O do not always occur in the preparation of BZ monomers due to low selectivity, which is strongly dependent on the substituent positions. In previous studies [27], we used the approach described by Ishida and Lin et al. to prepare BZ rings through a three-step synthesis (Scheme 1d–f). Here, we used the Schiff base formed from TAPP and salicylaldehyde to first form porphyrin-salicylaldehyde (Por-Sa) (Scheme 1d). Next, we reduced Por-Sa to form the *o*-hydroxybenzylamine derivative Por-Hy (Scheme 1e). Finally, the reaction of Por-Hy with the aldehyde derivative was provided the target monomer Por-BZ (Scheme 1f).

Figure 1 also presents FTIR spectra of Por-Sa, Por-Hy, and the monomer Por-BZ, recorded at room temperature. The O–H and N–H stretching absorptions appeared near 3455 and 3409 cm^−1^ for Por-Sa (Figure 1d) and Por-Hy (Figure 1e), respectively. After the reaction with paraformaldehyde, the signal of the OH groups was absent from the spectrum of the Por-BZ monomer, with the main characteristic signals being those for the oxazine ring (948 cm^−1^), C–O stretching (1222 cm^−1^), and N–H stretching of the pyrrole units (3319 cm^−1^), indicative of the BZ ring having formed. Figure 2 displays ^1^H and ^13^C NMR spectra of Por-Sa (Figure 2b), Por-Hy (Figure 2c), and Por-BZ monomer (Figure 2d) in DMSO-*d*_6_. The signals of the NH_2_ units of TAPP were absent in the spectrum of Por-Sa (Figure 2A-b), with characteristic signals appearing for the N=CH, OH, and NH units at 10.24, 10.72, and 8.90 ppm, respectively, along with signals for aromatic protons in the range 8.30–6.87 ppm. The spectrum of Por-Hy (Figure 2A-c) also featured signals for aromatic protons (7.88–6.87 ppm), but the signal of the N=CH units of Por-Sa (at 10.24 ppm) was disappeared, with the new signal for NHC**H**_2_ units appearing at 4.45 ppm, in addition to signals for the NH and OH units at 9.62 and 8.85 ppm, respectively. The high-field signal of the OH units was due to a change in intramolecular hydrogen bonding after the reduction of Por-Sa. The spectrum of Por-BZ (Figure 2A-d) featured signals for aromatic rings and NH units at 8.06–6.85 and 8.78 ppm, respectively. Furthermore, the signals of the oxazine ring protons were appeared at 4.93 (ArC**H**_2_N) and 5.72 (OCH_2_N) ppm with equal integral intensity. The corresponding ^13^C NMR spectra confirmed their chemical structures (Figure 2B). The ^13^C NMR spectrum of Por-Sa (Figure 2B-b) featured signals at 192.67, 161.75, and 137.02–117.54 ppm for the N=CH, COH, and aromatic carbon nuclei, respectively. The Por-Hy spectrum featured a new peak at 41.56 ppm for the NHCH_2_ carbon nuclei (Figure 2B-c). The characteristic signals for Por-BZ appeared at 49.16 (Ar**C**H_2_N) and 78.80 (OCH_2_N) ppm, indicating that the BZ ring had been synthesized with high purity and yield.

### 3.3. Thermal Curing Polymerization of Por-BZ Monomer

**Figure 3:**
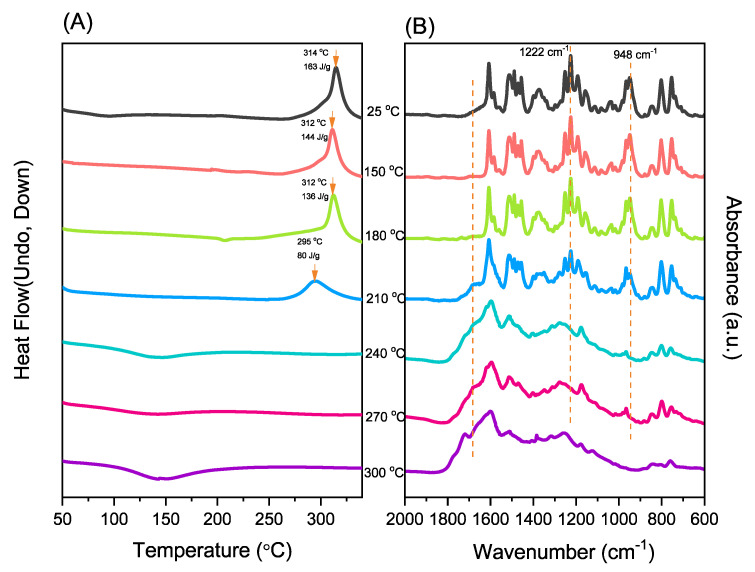
(**A**) DSC and (**B**) FTIR spectral analyses of the Por-BZ monomer after thermal ROP at various temperatures.

We used DSC, TGA, and FTIR spectroscopy to investigate the thermal curing polymerization of the Por-BZ monomer. Figure 3A displays DSC traces of pure Por-BZ monomer, which provided a high exothermic maximum curing temperature of 314 °C and the reaction heat of 163 J g^−1^. Compared with the typical thermal curing temperature of a Pa-type BZ monomer (255–263 °C), the significantly higher value for pure Por-BZ monomer was presumably because the rigid structure of its porphyrin unit inhibited ROP at relatively high temperatures. After thermal polymerizations at 150, 180, and 210 °C, the polymerization temperatures of Por-BZ shifted to 312, 312, and 295 °C, respectively, with the reaction heat of 144, 138, and 80 J g^−1^, respectively. In addition, after thermal curing polymerization at 240 °C for 2 h, no obvious exothermic peak was observed, suggesting that the complete ROP of the Por-BZ monomer had occurred at this temperature to form poly(Por-BZ). Figure 3B displays the FTIR spectra of Por-BZ after its thermal polymerizations at the various temperatures. The signals of BZ units at 1222 and 948 cm^−1^ decreased gradually upon increasing the thermal polymerization temperature, consistent with ring-opening of the oxazine structure. After the temperature had increased to 240 °C, the signals of the oxazine ring disappeared completely based on the FTIR spectra, suggesting the formation of the highly cross-linked poly(Por-BZ) structure (Scheme S1), which is consistent with DSC data.

**Figure 4:**
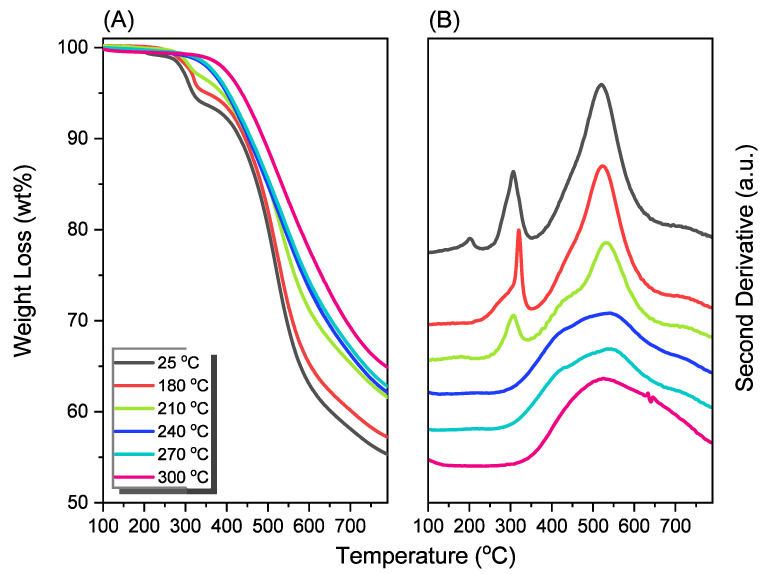
(**A**) TGA and (**B**) corresponding second-derivative curves based on TGA analyses of the Por-BZ monomer after thermal ROP at various temperatures.

Furthermore, we used TGA to investigate the thermal properties of Por-BZ monomer after thermal ROP at different temperatures. Figure 4A shows that the thermal decomposition temperatures (*T*_d10_) for the Por-BZ monomer thermally cured at 25, 180, 210, 240, 270, and 300 °C were 434, 445, 453, 454, 460, and 478 °C, respectively; the corresponding char yields were 55, 57, 61, 62, 63, and 66 wt%, respectively. The *T*_d10_ values and char yields of this new poly(Por-BZ) were higher than those of traditional BZs (typically 405 °C and 35 wt%, respectively), presumably because of the presence of highly aromatic structures. The *T*_d_ and char yield were both increased with the increase in thermal ROP temperature, suggesting structures of higher cross-linking density. Figure 4B reveals that the exothermic peaks of Por-BZ near 300 °C disappeared gradually with the increase in the thermal polymerization temperature, and were completely absent after thermal treatment at 240 °C, consistent with the DSC and FTIR results in Figure 3.

### 3.4. Characterization of Por-BZ/Metal Complex

**Figure 5:**
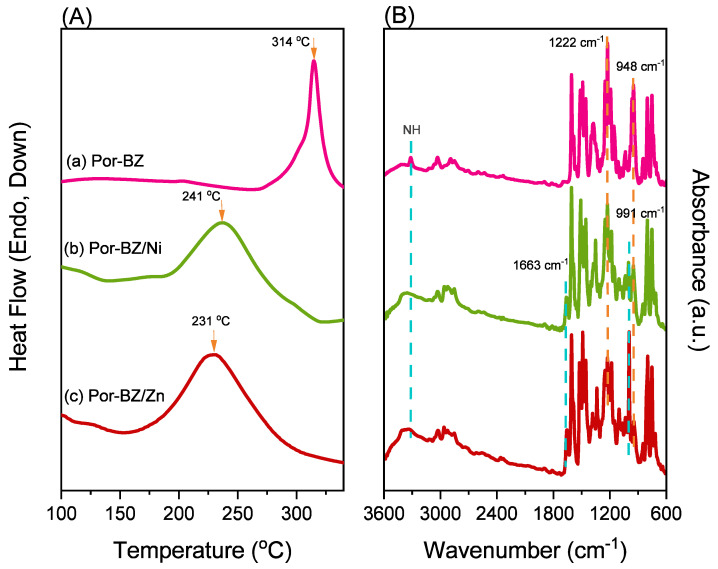
(**A**) DSC and (**B**) FTIR spectral analyses of the (**a**) Por-BZ monomer, (**b**) Por-BZ/Ni complex, and (**c**) Por-BZ/Zn complex prior to thermal ROP.

Because porphyrins are excellent moieties for complexing metal cations, we tested the ability of our Por-BZ to bind Ni^2+^ and Zn^2+^ ions [52,53]. We used DSC and FTIR spectroscopy to demonstrate the incorporation of these metal ions into the porphyrin structures, and DSC thermograms to record the thermal ROP behavior of the Por-BZ/Ni and Por-BZ/Zn complexes. Figure 5A presents that the thermal curing peaks of the uncured Por-BZ/Ni and Por-BZ/Zn complexes were centered at 241 and 231 °C, respectively, implying that the metal ions could act as catalysts for the ROPs of the BZ rings. In previous studies we found that the mechanism through which these metal ions catalyze the ring-opening of BZ involves three steps: first, the metal ion coordinates the nitrogen or oxygen atom in the oxazine ring structure, and then the next electrophilic attack of the metal ions to the oxazine ring occurs. Finally, the rearrangement was occurred to form the stable phenolic and phenoxy structures [3]. Figure 5B presents FTIR spectra of Por-BZ and the Por-BZ/Ni and Por-BZ/Zn complexes, measured at room temperature. The BZ structure (signals at 1222 and 948 cm^−1^) remained for the uncured Por-BZ/Ni and Por-BZ/Zn complexes. Meanwhile, the signal at 3316 cm^−1^ for NH stretching of the pyrrole ring had disappeared, suggesting that the metal ions had been inserted into the porphyrin structure, replacing the original pyrrole protons (Scheme 2b), with new signals observed at 1663 and 991 cm^−1^ in the FTIR spectra of the Por-BZ/Ni and Por-BZ/Zn complexes, arising from coordination of the metal ions with the porphyrin structures.

**Figure 6:**
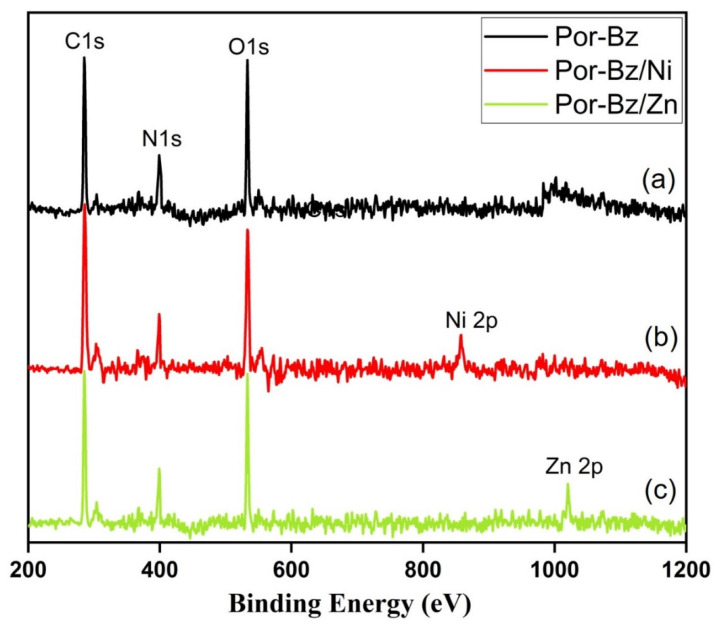
XPS spectra of the (**a**) Por-BZ monomer, (**b**) Por-BZ/Ni complex, and (**c**) Por-BZ/Zn complex.

**Figure 7:**
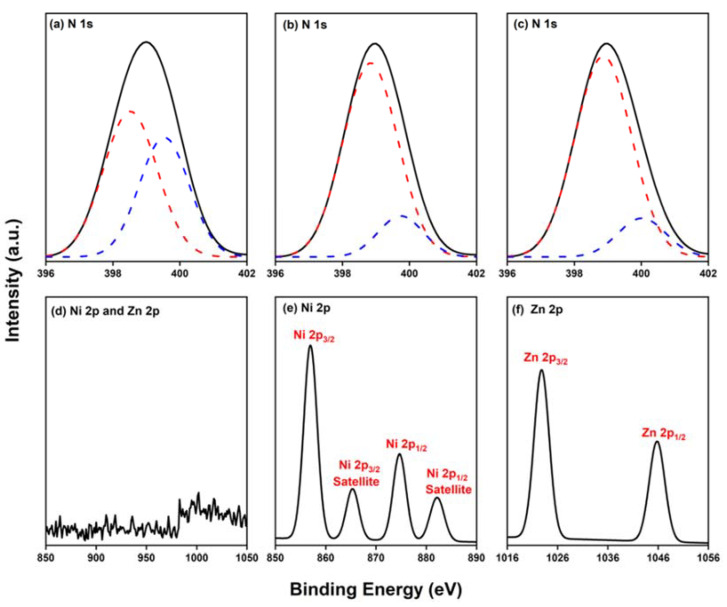
XPS spectra of the N1s species (**a**) Por-BZ monomer, (**b**) Por-BZ/Ni complex, and (**c**) Por-BZ/Zn complex. XPS spectra of (**d**) the Ni 2p, and Zn 2p species of Por-BZ monomer, (**e**) the Ni 2p species of Por-BZ/Ni complex, and (**f**) the Zn 2p species of Por-BZ/Zn complex.

The incorporation of Ni^2+^ and Zn^2+^ ions into the porphyrin structures was futher confirmed using X-ray photoelectron spectroscopy (XPS). As observed in Figure 6, the Por-BZ exhibited three peaks at 285.27, 398.58, and 533.56 eV for the carbon, nitrogen, and oxgyen atoms, respectively. The Por-BZ/Ni complex featured four charatersitic peaks at 285.27, 399.95, 534.70, 858.45 eV for the carbon, nitrogen, oxgyen, and nickel atoms, respectively, while the Por-BZ/Zn complex exhibited four peaks at 285.28, 399.94, 533.96, and 1021.02 eV for carbon, nitrogen, oxgyen, and zinc atoms, respectively. The apperance of nickel and zinc peaks confirmed the successful incorporation of metal ions in the porphyrin units. To have a better understanding of the different types of N and metal species found in Por-BZ, and the Por-BZ/Ni and Por-BZ/Zn complexes, we fitted their XPS peaks for the N 1s, Ni 2p, and Zn 2p orbitals (Figure 7). Figure 7a revealed that the free-base porphyrin Por-Bz had two different types of N 1s species: iminic nitrogen (=N–) at 398.50 eV and aminic nitrogen (–NH–) at 399.53 eV. The area fractions of iminic and aminic nitrogen were found to be 57 and 43%, respectively. On the other hand, Por-BZ/Ni and Por-BZ/Zn complexes possessed two types of N 1s species at 398.88 and 398.87 eV for iminic nitrogens, respectively, and at 399.77 and 399.98 eV for aminic nitrogens, respectively (Figure 7b,c). The area fractions of iminic and aminic nitrogen were found to be 86 and 14%, respectively, and for the Por-BZ/Ni complexe, 87 and 13%, respectively. The strong decreasing of the peaks of aminic nitrogens in Por-BZ/Ni and Por-BZ/Zn complexes confirmed the metal ions had been incorporated into the porphyrin structure, taking the place of the original pyrrole protons. Futhermore, the Por-BZ did not show the metal ion peaks (Figure 7d), while the fitting of XPS peaks for the Ni 2p, and Zn 2p orbitals revealed that the Por-BZ/Ni complex had four Ni 2p species: Ni 2p_3/2_ (857.88 eV), Ni 2p_3/2_ satellite (865.43 eV), Ni 2p_1/2_ (874.79 eV), and Ni 2p_1/2_ satellite (882.25 eV) (Figure 7e). The Por-BZ/Zn complex had two Zn 2p species: Zn 2p_3/2_ (1022.91 eV) and Zn 2p_1/2_ (1045.75 eV) (Figure 7f).

**Figure 8:**
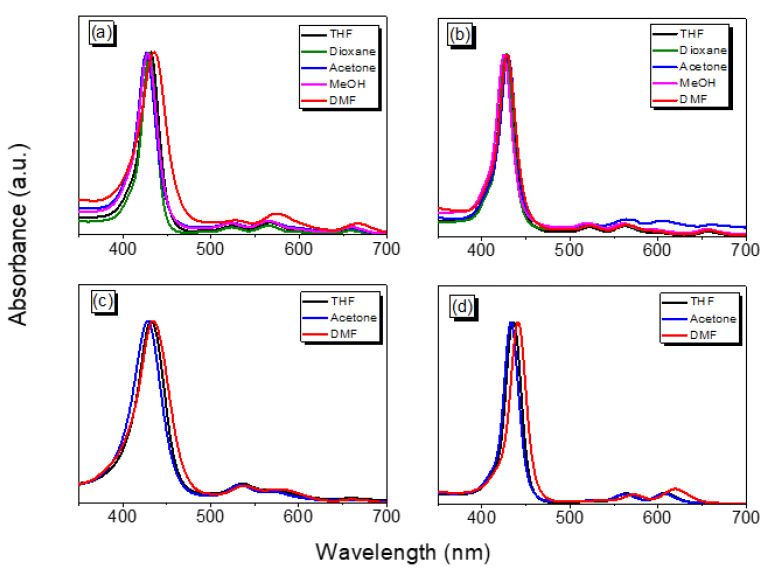
UV–Vis absorption spectra of (**a**) TAPP, (**b**) Por-BZ, (**c**) the Por-BZ/Ni complex, and (**d**) the Por-BZ/Zn complex.

To study the luminescence properties of TAPP, Por-BZ, and the Por-BZ/Ni and Por-BZ/Zn complexes in various solvents, we recorded their UV–Vis absorption spectra at room temperature (Figure 8, Table 1). The highly conjugated porphyrin structure usually exhibits the following features in its UV–Vis absorption spectrum: a very strong absorption band near 400 nm, named the Soret band, resulting from the transition of electrons from the S_0_ to S_2_ state; and several weaker signals between 450 and 700 nm, corresponding to the Q band and representing electronic transitions from S_0_ to S_1_ [54]. The main peaks of soret bands and Q bands of our products were shown in Table 1. Placing exterior substituents on the porphyrin ring usually induces minor changes in the wavelengths and intensities of the absorption bands, while protonation of the two inner nitrogen atoms or the insertion (or change) of a metal atom at the core of the porphyrin generally has a dramatic effect on the visible absorption spectrum [54]. Compared with the absorption bands of TAPP (Figure 8a), the absorption bands of Por-BZ (Figure 8b) were slightly blue-shifted within 8 nm, whereas the addition of the metal ions caused the bands to red-shift dramatically. After metalation, the peaks of Soret bands red-shifted about 15 nm from Por-BZ to Por-BZ/Ni, and about 40 nm for Por-BZ/Zn; meanwhile Q bands red-shifted about 6 nm after adding Ni^2+^ ions and 12 nm for Zi^2+^, relatively. We suspect that the electron-donating units on the phenyl groups at the meso positions of the porphyrin ring enhanced the electron density of the phenyl ring, causing the phenyl ring to conjugate with the porphyrin macrocycle to a certain degree. This kind of conjugation would likely lower the electron transition energy of the porphyrin macrocycle, resulting in red-shifts of the absorption bands. After transforming the NH_2_ units to BZ rings, this interaction was inhibited, causing the adsorption bands to be blue-shifted. After bonding to the central nitrogen atoms of a porphyrin, metal ions accept the lone pairs of electrons of the N atoms of the pyrrole rings, with the metal ions donating electrons to the porphyrin ring to induce delocalized π-bonds, which permit the ready flow of electrons within the delocalized π-system. In this study, after adding metal ions (Figure 8c,d), the number of Q bands decreased and their absorption frequencies shifted. When the metal ions coordinated with porphyrin ligand, the symmetry of the molecule changed from D_2h_ to D_4h_, with the cleavage degree of the molecular orbital decreasing and the degeneracy increasing; hence, the number of Q bands decreased [55,56].

**Figure 9:**
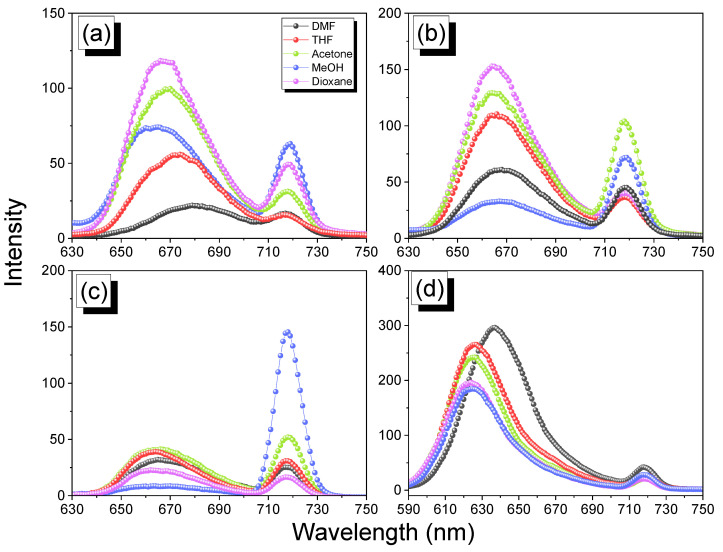
PL spectra of (**a**) TAPP, (**b**) Por-BZ, (**c**) the Por-BZ/Ni complex, and (**d**) the Por-BZ/Zn complex.

We also recorded PL spectra to measure the fluorescence properties of TAPP, Por-BZ, and the Por-BZ/Ni and Por-BZ/Zn complexes dissolved in several kinds of solvents (Figure 9). Fluorescent materials with highly π-conjugated systems typically exhibit strong luminescence in dilute solution; their fluorescence emissions are typical quenched in solutions of high concentrations or in the solid state, a phenomenon known as “aggregation-caused quenching” (ACQ), a result of strong face-to-face π-stacking [57,58,59,60,61]. Therefore, we investigated the effect of concentration on the fluorescence emissions of TAPP and Por-BZ in DMF upon excitation at a wavelength of 360 nm. Figure S1 reveals that the emission intensity decreased upon increasing the concentrations of both TAPP and Por-BZ in DMF, confirming their ACQ behavior. Furthermore, we investigated the PL behavior of TAPP, Por-BZ, and the Por-BZ/Ni and Por-BZ/Zn complexes by recording their fluorescence emissions when dispersed in various solvents. In general, we prepared solutions of our powder products dissolved in the various solvents at a concentration of 10^–4^ mol L^−1^. It was relatively difficult to dissolve Por-BZ and the Por-BZ/Zn complex in MeOH; Porphyrin-BZ-Ni formed suspensions in these organic solvents. The emission spectra are presented in Figure 7, after excitation at a wavelength of 360 nm. According to previous reports [55,62], the typical emission spectrum for a porphyrin shows two peaks centered at approximately 650 and 720 nm, due to the Q (0-0) and the Q (0-1) transitions, respectively. Excitation of our porphyrin derivatives to the S2 (B band) and S1 (Q band) levels resulted in fluorescence, with the fluorescence of the S2 (B band)—representing the transition from the second excited singlet state S2 to the ground state S0 (S2→S0)—corresponding to the Soret band in the UV–Vis absorption spectra. The fluorescence of the Q band is corresponding to the transition from the lowest excited singlet state S1 to the ground state S0. The fluorescence of S2→S0 was too weak to be observed in this study, owing to the light scattering and resorption of the strong Soret absorption bands. Interestingly, compared with the fluorescent bands of TAPP and Por-BZ, the emission peaks of the Por-BZ/Zn complex were much stronger and had been blue-shifted significantly, from 665 nm to 630 nm. Nevertheless, the PL intensity of the Por-BZ/Ni complex was lower than that of Por-BZ, with no shifts of the peaks. Thus, the addition of different kinds of metal ions had dramatically different impacts on the fluorescence properties of Por-BZ.

### 3.5. Thermal ROP of Por-BZ/Metal Complex

**Figure 10:**
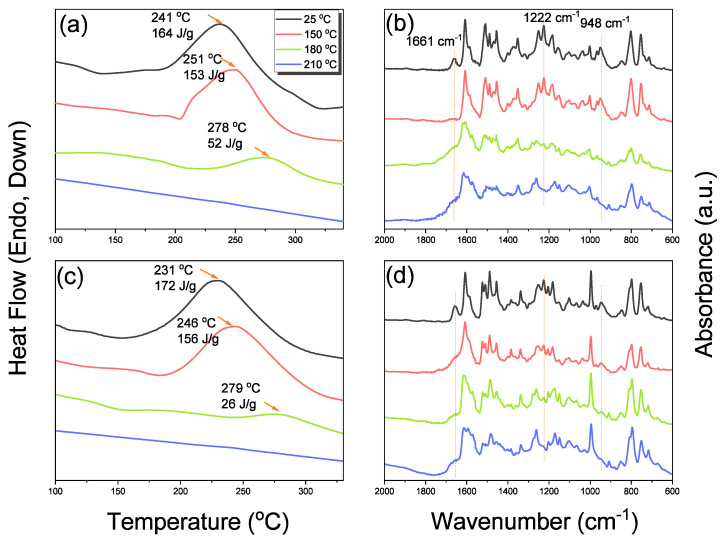
DSC and FTIR spectral analyses of (**a**,**b**) Por-BZ/Ni and (**c**,**d**) Por-BZ/Zn complexes after thermal ROP at various temperatures.

**Figure 11:**
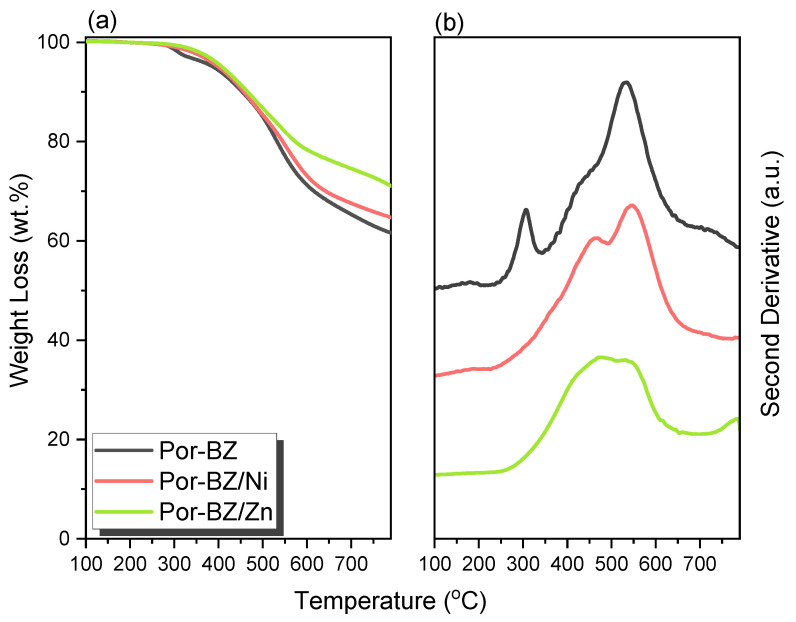
(**a**) TGA and (**b**) corresponding second-derivative curves based on TGA analyses of the Por-BZ monomer and the Por-BZ/Ni and Por-BZ/Zn complexes after thermal ROP at 210 °C.

We used DSC analyses to monitor the thermal ROP behavior of Por-BZ/Ni and Por-BZ/Zn complexes after their thermal curing for 2 h at temperatures of 150, 180, and 210 °C, respectively. Figure 10a,c reveal that the thermal curing peak temperatures of uncured Por-BZ/Ni and Por-BZ/Zn complexes were centered at 241 and 231 °C, respectively, as mentioned in our discussion of Figure 5A. Similar to the behavior of pure Por-BZ, the exothermic enthalpies of both the Por-BZ/Ni and Por-BZ/Zn complexes decreased with the increase in temperature, and disappeared completely after thermal treatment at 210 °C. Although the thermal curing behavior was consistent with that of the pure Por-BZ under the same thermal curing conditions, which was much lower than that of pure Por-BZ monomer, suggesting that the presence of the Ni and Zn ions assisted the ring-opening procedure. We used FTIR to study the thermal curing polymerization behavior of Por-BZ/Ni and Por-BZ/Zn complexes after thermal polymerization at different temperatures (Figure 10b,d). Similar to Figure 3B, when the thermal polymerization temperature was increased, the characteristic absorption bands of the oxazine structure at 1222 and 948 cm^−1^ gradually decreased, and disappeared completely as the thermal curing temperature was only 210 °C, compared with 240 °C for the pure Por-BZ monomer, again suggesting that the Ni (Figure 10b) and Zn (Figure 10d) ions assisted the ROP, and implying the formation of highly cross-linked poly(Por-BZ/metal) complexes. Figure 11a displays the results of TGA analyses of pure Por-BZ and the Por-BZ/Ni and Por-BZ/Zn complexes after thermal ROP at 210 °C. The values of *T*_d10_ and the char yields increased to 458 °C and 64.3 wt%, respectively, for the poly(Por-BZ/Ni) complex and to 465 °C and 71.1 wt%, respectively, for the poly(Por-BZ/Zn) complex, when compared with those of the pure poly(Por-BZ) (453 °C and 61.5 wt%, respectively). Figure 9B displays these three corresponding exothermic peaks after thermal ROP at 210 °C; the first exothermic peaks disappeared for both the Por-BZ/Ni and Por-BZ/Zn complexes, indicating that the Ni and Zn ions not only facilitated the ROP of Por-BZ, but also enhanced the thermal stability and char yield significantly based on TGA analyses. According to the DSC, FTIR spectral, and TGA analyses, the coordination ability to the porphyrin structure of the Zn ion appeared to be stronger than that of the Ni ion, as has been discussed widely in previous reports [49], resulting in a lower ROP temperature and a higher thermal stability.

## 4. Conclusions

We synthesized the new porphyrin-functionalized BZ monomer from the reaction of TAPP with salicylaldehyde, and the subsequent reduction of the Schiff base of Por-Sa with CH_2_O, with characterization based on NMR and FTIR spectroscopy. Because of the rigid porphyrin structure, the resulting poly(Por-BZ) displayed excellent thermal stability, with the thermal exothermic curing peak of Por-BZ shifted to 314 °C. After the addition of Ni and Zn ions, however, this thermal exothermic curing peak shifted significantly to 241 and 231 °C, respectively, suggesting that these metal ions accelerated the ROP of the Por-BZ unit. Furthermore, the thermal stability of these Por-BZ/metal complexes improved, compared with that of the pure Por-BZ, after thermal polymerization.

## Figures and Tables

**Table 1:** The main peaks in the UV/Vis spectra of TAPP, Por-BZ, Por-BZ/Ni and Por-BZ/Zn.

Compound	Solvent	λ_abs_ (nm) (Soret Band)	λ_abs_ (nm) (Q Band)
TAPP	THF	524, 566, 602, 662	432
	Dioxane	524, 564, 600, 660	430
	Acetone	522, 564, 600, 660	428
	MeOH	522, 564, 596, 656	426
	DMF	528, 574, 608, 666	436
Por-BZ	THF	522, 562, 598, 658	428
	Dioxane	522, 562, 598, 656	428
	Acetone	520, 560, 596, 654	424
	MeOH	522, 564, 606, 662	430
	DMF	522, 564, 600, 658	428
Por-BZ/Ni	THF	535, 574, 657.5	432
	Acetone	533.5, 571, 656.5	428
	DMF	538, 579, 660.5	434
Por-BZ/Zn	THF	564, 608	436
	Acetone	562, 606	432
	DMF	572, 620	440

## Data Availability

Not applicable.

## Supplementary Materials

The following are available online at https://www.mdpi.com/article/10.3390/polym14030449/s1, Scheme S1: the chemical structure of Por-BZ and their thermal polymerization, Figure S1: PL profile of TAPP and Por-BZ/Zn complex.
