# Supplementary material for: Metal Complexes of the Porphyrin-Functionalized Polybenzoxazine
Source: Polymers (Basel). 2022 Jan 23;14(3):449. doi: 10.3390/polym14030449 (PMC8839356; doi:10.3390/polym14030449)
Supplement: Supplementary file 1 [file polymers-14-00449-s001.zip › polymers-1525717-supplementary.pdf]

Supporting Information

Metal Complexes of the Porphyrin-functionalized Polybenzoxazine

Guohu Zhang [1], Ahmed F. M. EL-Mahdy [1], Lamiaa Reda Ahmed [1,2], Babasaheb M. Matsagar [3], Sameerah Al-Saeedi [4], Shiao-Wei Kuo [1,5*] and Kevin C.-W. Wu [3,6*]

1 Department of Materials and Optoelectronic Science, Center for Functional Polymers and Supramolecular Materials, National Sun Yat-Sen University, Kaohsiung 80424, Taiwan; m083100049@student.nsysu.edu.tw (G. Z.); ahmedelmahdy@mail.nsysu.edu.tw (A. F. M. E); kuosw@faculty.nsysu.edu.tw (S.-W.K.)

2 Institute of Medical Science and Technology, National Sun Yat-Sen University, Kaohsiung 80424, Taiwan; d102060004@student.nsysu.edu.tw (L. R. A.)

3 Department of Chemical Engineering, National Taiwan University, No. 1, Sec. 4, Roosevelt Road, Taipei 10617, Taiwan; matsagar03@ntu.edu.tw (B M. M.); kevinwu@ntu.edu.tw (K. C. W. W.)

4 Department of Chemistry, College of Science, Princess Nourah Bint Abdulrahman University, Riyadh 11671, Saudi Arabia; sialsaeedi@pnu.edu.sa (S. A.)

5 Department of Medicinal and Applied Chemistry, Kaohsiung Medical University, Kaohsiung 80708, Taiwan; kuosw@faculty.nsysu.edu.tw (S.-W.K.)

6 International Graduate Program of Molecular Science and Technology, National Taiwan University (NTU-MST), No. 1, Sec. 4, Roosevelt Road, Taipei 10617, Taiwan

* Correspondence: kuosw@faculty.nsysu.edu.tw (S.-W. K.); kevinwu@ntu.edu.tw (K. C.-W. W.)

Characterization

FTIR spectra were recorded using a Bruker Tensor 27 FTIR spectrophotometer and the conventional KBr plate method; 32 scans were collected at a resolution of 4 cm^{-1}. ^{1}H and ^{13}C NMR spectra were recorded using an INOVA 500 instrument with DMSO-d_6 and $CDCl_3$ as solvents and tetramethylsilane (TMS) as the external standard. Chemical shifts are provided in parts per million (ppm). DSC analyses were performed using TA Q-20 differential scanning calorimeter operated under a N_2 atmosphere. The sample (ca. 3-5 mg) was placed in a sealed aluminum sample pan from 40 to 350 ℃ at a heating rate of 20 °C/min. TGA was performed using a TA Q-50 analyzer under a flow of N2. The samples were sealed in a Pt cell and heated from 40 to 800 °C at a heating rate of 20 °C min–1 under N2 at a flow rate of 50 mL min^{-1}. UV–Vis absorption spectra were recorded using an F-4500 fluorescence spectrometer. Fluorescence emission spectra were recorded using a LabGuide X350 spectrometer.

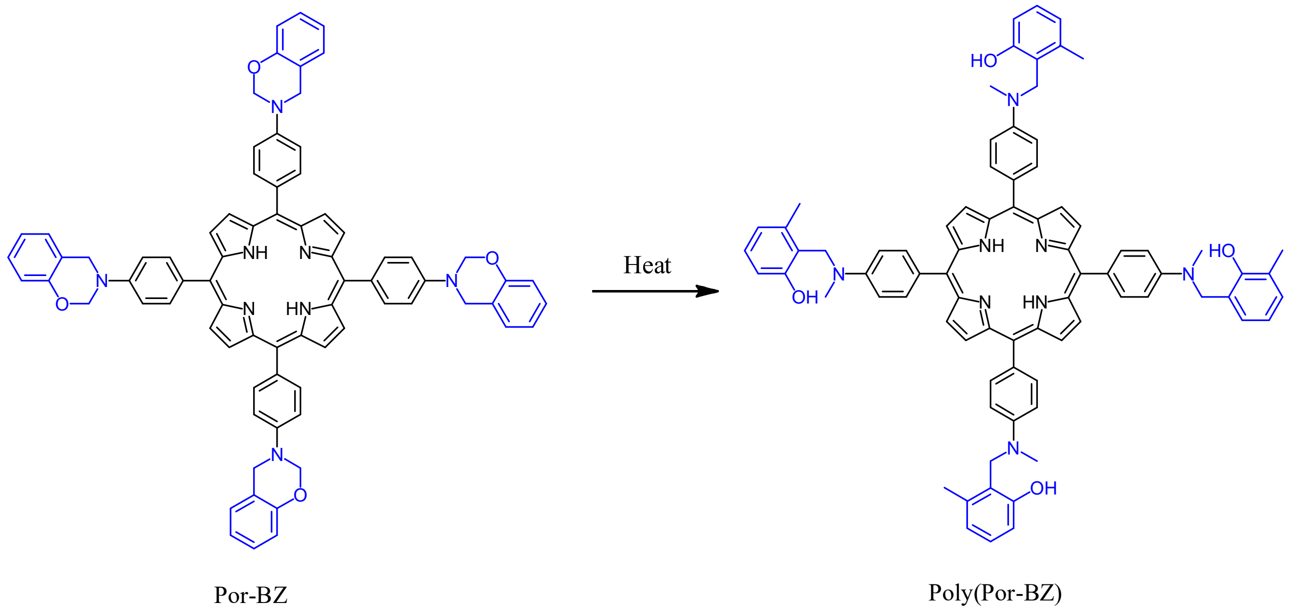

Scheme S1: The chemical structure of Por-BZ after thermal ring opening polymerization

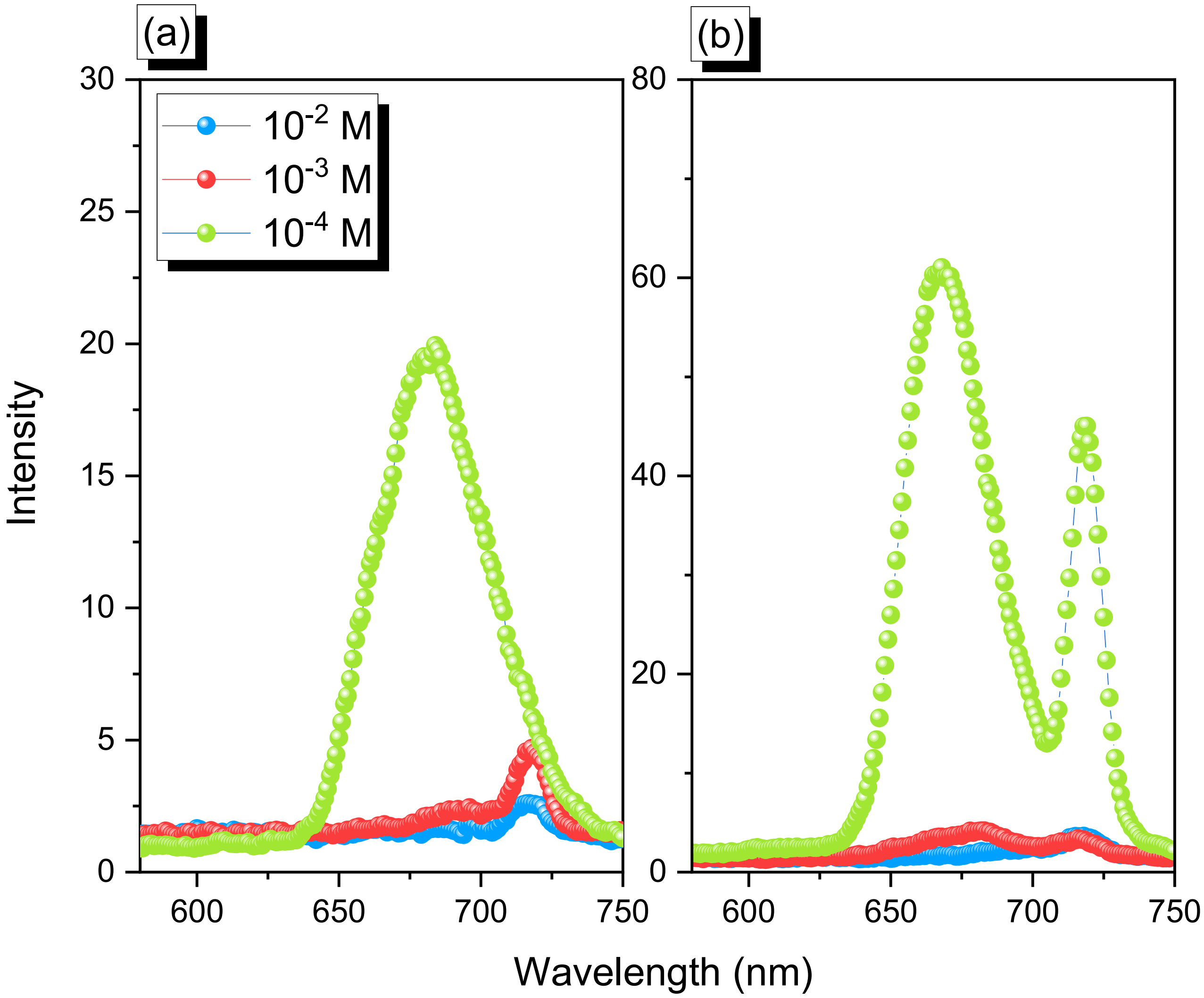

Figure S1: PL spectra of (a) TAPP, (b) Por-BZ/Zn complex recorded at room temperature
